# Potential of Plant-Based Extracts to Alleviate Sorbitol-Induced Osmotic Stress in Cabbage Seedlings

**DOI:** 10.3390/plants13060843

**Published:** 2024-03-14

**Authors:** Katarzyna Pacyga, Paweł Pacyga, Aleksandra Boba, Bartosz Kozak, Łukasz Wolko, Yelyzaveta Kochneva, Izabela Michalak

**Affiliations:** 1 Department of Environment Hygiene and Animal Welfare, Faculty of Biology and Animal Science, Wrocław University of Environmental and Life Sciences, 50-375 Wrocław, Poland; 2 Department of Thermodynamics and Renewable Energy Sources, Faculty of Mechanical and Power Engineering, Wrocław University of Science and Technology, 50-370 Wrocław, Poland; pawel.pacyga@pwr.edu.pl; 3 Department of Genetic Biochemistry, Faculty of Biotechnology, University of Wrocław, 51-148 Wrocław, Poland; aleksandra.boba@uwr.edu.pl (A.B.); yelyzaveta.kochneva@uwr.edu.pl (Y.K.); 4 Department of Genetics, Plant Breeding and Seed Production, Faculty of Life Sciences and Technology, Wrocław University of Environmental and Life Sciences, 50-363 Wrocław, Poland; bartosz.kozak@upwr.edu.pl; 5 Department of Biochemistry and Biotechnology, Faculty of Agriculture, Horticulture and Bioengineering, Poznan University of Life Sciences, 60-632 Poznań, Poland; lukasz.wolko@up.poznan.pl; 6 Department of Advanced Material Technologies, Faculty of Chemistry, Wrocław University of Science and Technology, 50-372 Wrocław, Poland; izabela.michalak@pwr.edu.pl

**Keywords:** plant-based extracts, prosperous biostimulants, Petri dish tests, foliar spray, sorbitol-induced stress, chemical analysis, gene expression

## Abstract

In light of expected climate change, it is important to seek nature-based solutions that can contribute to the protection of our planet as well as to help overcome the emerging adverse changes. In an agricultural context, increasing plant resistance to abiotic stress seems to be crucial. Therefore, the scope of the presented research was focused on the application of botanical extracts that exerted positive effects on model plants growing under controlled laboratory conditions, as well as plants subjected to sorbitol-induced osmotic stress. Foliar spraying increased the length and fresh mass of the shoots (e.g., extracts from *Taraxacum officinale*, *Trifolium pratense*, and *Pisum sativum*) and the roots (e.g., *Solidago gigantea*, *Hypericum perforatum*, and *Pisum sativum*) of cabbage seedlings grown under stressful conditions, as well as their content of photosynthetic pigments (*Pisum sativum*, *Lens culinaris*, and *Hypericum perforatum*) along with total phenolic compounds (*Hypericum perforatum*, *Taraxacum officinale*, and *Urtica dioica*). The antioxidant activity of the shoots measured with the use of DDPH (*Pisum sativum*, *Taraxacum officinale*, *Urtica dioica*, and *Hypericum perforatum*), ABTS (*Trifolium pratense*, *Symphytum officinale*, *Valeriana officinalis*, *Pisum sativum*, and *Lens culinaris*), and FRAP (*Symphytum officinale*, *Valeriana officinalis*, *Urtica dioica*, *Hypericum perforatum*, and *Taraxacum officinale*) assays was also enhanced in plants exposed to osmotic stress. Based on these findings, the most promising formulation based on *Symphytum officinale* was selected and subjected to transcriptomic analysis. The modification of the expression of the following genes was noted: *Bol029651* (glutathione S-transferase), *Bol027348* (chlorophyll A-B binding protein), *Bol015841* (S-adenosylmethionine-dependent methyltransferases), *Bol009860* (chlorophyll A-B binding protein), *Bol022819* (GDSL lipase/esterase), *Bol036512* (heat shock protein 70 family), *Bol005916* (DnaJ Chaperone), *Bol028754* (pre-mRNA splicing Prp18-interacting factor), *Bol009568* (heat shock protein Hsp90 family), *Bol039362* (gibberellin regulated protein), *Bol007693* (B-box-type zinc finger), *Bol034610* (RmlC-like cupin domain superfamily), *Bol019811* (myb_SHAQKYF: myb-like DNA-binding domain, SHAQKYF class), *Bol028965* (DA1-like Protein). Gene Ontology functional analysis indicated that the application of the extract led to a decrease in the expression of many genes related to the response to stress and photosynthetic systems, which may confirm a reduction in the level of oxidative stress in plants treated with biostimulants. The conducted studies showed that the use of innovative plant-based products exerted positive effects on crops and can be used to supplement current cultivation practices.

## 1. Introduction

It is expected that climate change will increase the occurrence of unfavourable environmental conditions for crop cultivation around the world. Furthermore, as these alterations continue in the future, substantial areas of high-quality agricultural land will presumably be destroyed by erosion, desertification, rising seas, and salinisation [1]. Moreover, agricultural production is also under threat due to the increasing prevalence of diseases and pests [2] and the unbalanced usage of mineral fertilisers and pesticides [3]. The level of food production needs to be increased, regardless of the smaller available area for farming and more severe conditions for plant growth [4]. Therefore, novel approaches are needed to maintain the food, fibre, and fuel requirements of an increasing world population, which is expected to reach 9.7 billion in 2050 [5].

This goal may prove to be an exceedingly challenging task as, during growth, plants are exposed to biotic and abiotic stresses, which can be of natural or anthropogenic origin [6]. It has been estimated that adverse environmental effects caused by climate change have reduced crop yields by up to 70% since 1982 [7]. Stress significantly affects the biochemical, morphological, and physiological mechanisms of plants, along with gene regulation [8]. In response to unfavourable conditions, a complex range of responses is triggered, which includes various physiological pathways of primary and secondary metabolism [9]. For instance, under stressful conditions, the level of reactive oxygen species (ROS), which originate from oxidation processes (like photosynthesis and respiration), substantially rises and may result in damage to lipid membranes, nucleic acids, and proteins [10]. To overcome the stress conditions and ROS assemblage, plants have evolved several mechanisms, which include the accumulation of ascorbic acid, carotenoids, flavonoids, glucosinolates, osmolytes, specific proteins, and sugars and the activation of hormone-mediated responsive networks that involve jasmonates and other signalling molecules [11].

In addition to a sufficient amount of food, it is also essential to ensure that plant-based products are of high quality [12] and free of synthetic chemical residues that can induce detrimental health effects [13]. Nowadays, consumer awareness and requirements for food quality and safety are constantly growing, which simultaneously impose the necessity of substituting synthetic chemicals with products based on natural derivatives, called biostimulants [14]. A plant biostimulant is defined as: “*any substance or microorganism applied to plants with the aim to enhance nutrition efficiency, abiotic stress tolerance and/or crop quality traits, regardless of its nutrients content*” [15]. These preparations are a rich source of bioactive compounds (e.g., acids, antioxidants, hormones, fats, minerals, oils, pigments, polysaccharides, and vitamins) which exhibit diverse activities [16]. They are usually applied in the form of seed priming or coating, foliar spray, or root dipping and/or direct applications to the soil [17]. Treatment with biostimulants benefits crop cultivation by improving germination rates, seedling vigour, nutrient uptake, growth, development, and plant metabolism, as well as increasing tolerance to biotic and abiotic stresses [18]. In recent decades, a tremendous increase in their use has been observed [19]. This is evidenced by the fact that the global biostimulant market is assessed at USD 3 billion in 2021 and is expected to exceed more than USD 5.1 billion by 2027 [20]. Therefore, understanding their mechanism of action is crucial and often necessitates a multidisciplinary approach due to the plethora of interactions between a great variety of bioactive compounds within the same extract [21]. For this reason, within the scope of the conducted research, our aim was to discover the mechanism of action of potential biostimulants that exerted favourable effects on plants growing under controlled laboratory conditions, as well as plants subjected to osmotic stress by sorbitol. Most of the adverse growth conditions enforce osmotic stress on plants by reducing the water potential of the environment [22]. In our research, one of the most frequently found polyols in plants, namely sorbitol, was used to induce stress. This sugar is a direct product of photosynthesis in leaves and serves vital functions, such as the translocation of carbon skeletons and energy between sources and sink organs. The enhanced transport of polyols in the xylem and phloem occurs as a result of salt or drought stress [23]. Understanding plant responses to various unfavourable growth conditions and the underlying mechanism of action of biostimulating products is crucial for the development of innovative approaches to minimise the adverse effects of mineral fertilisers and plant protection products, as well as for progress in the individualisation of crop production. This is the first work that presents the effects of the application of botanical extracts on plants growing under stress conditions and attempts to decipher their mechanism of action under controlled laboratory conditions.

## 2. Results

The findings indicated that extracts derived from plants commonly occurring in the natural environment, often perceived as weeds, significantly affected the length and mass, as well as the content of photosynthetic pigments and total phenolic compounds, along with the antioxidant activity of cabbage seedlings. The conducted transcriptomic analysis contributed to the elucidation of potential mechanisms of action. The abbreviations for the raw materials utilised for the production of extracts used throughout this paper are as follows: Ur L—*Urtica dioica* L. (leaves), Sg L—*Solidago gigantea* Aiton (leaves), To F—*Taraxacum officinale* F.H. Wiggers coll. (flowers), To L—*Taraxacum officinale* F.H. Wiggers coll. (leaves), Tp F—*Trifolium pratense* L. (flowers), Vo R—*Valeriana officinalis* L. (roots), Hp H—*Hypericum perforatum* L. (herb), So R—*Symphytum officinale* L. (roots), Ps S—*Pisum sativum* L. (seeds), and Lc S—*Lens culinaris* Medik. (seeds).

### 2.1. Biometric Measurements

Under controlled laboratory conditions (Table 1), the application of extracts positively influenced the length of shoots and roots of cabbage seedlings. The longest above-ground parts were observed in groups treated with extracts based on So R (22.0% and 16.9% longer than in C—control and CB—commercial biostimulant, respectively) and Ur L (19.5% and 14.5% longer than in C and CB, respectively). In contrast, the shortest above-ground parts were noted only after the application of Hp H (10.7% and 14.5% shorter than in C and CB, respectively). On the other hand, in the case of roots, extracts obtained from To L, Tp F, and To F resulted in the lengthening of the root system by 23.8%, 21.1%, and 19.1% in comparison to C and by 24.2%, 21.5%, and 19.5% in relation to CB. However, the use of the Lc S-based extract showed the least growth-stimulating activity of the root system (no statistically significant difference).

It can also be observed that sorbitol-induced stress affected the growth of the model plant (Table 1). In most cases, foliar spraying with extracts enabled the plants to grow longer despite adverse growth conditions, in contrast to plants that were treated with water alone. A similar correlation was observed between the experimental groups and the group treated with a commercial biostimulant (excluding the length of plant shoots subjected to 300 mM sorbitol). For example, the application of extracts based on To L increased the shoot length by 24.1% and 14.4% under the lowest concentration of sorbitol (compared to C and CB, respectively), and extracts from Tp F increased the shoot length by 16.5% and 10.2% under 200 mM of sorbitol (compared to C and CB, respectively), while extracts obtained from To F increased the shoot length by 21.4% under 300 mM of sorbitol (compared to C). The least stimulating activity was exhibited by Hp H. In these groups, shoot length decreased by 16.3% and 22.9% under treatment with 100 mM of sorbitol, 15.8% and 20.4% under 200 mM of sorbitol compared to C and CB, respectively, and increased by 1.8% and decreased by 20.8% under 300 mM of sorbitol in comparison to C and CB, respectively. The use of extract based on Sg L increased the length of roots by 19.7%, 20.1%, and 43.8% compared to plants from the control group (C) grown under increasing sorbitol concentrations, respectively. In comparison to CB, the observed increase was 15.4%, 19.6%, and 20.9%, respectively. The Ur L-based extract was the only product that significantly reduced root length (by 24.9% and 36.8%) and only under conditions of the highest sorbitol concentration compared to C and CB, respectively.

In most cases, similarly to plant length, their fresh mass (FM) was also higher in the experimental groups under controlled laboratory conditions (Table 2). Extracts based on Lc S, Ps S, So R, and Ur L increased the fresh mass of shoots to the greatest extent—by 61.4%, 55.7%, 54.5%, and 51.1% in comparison to C, respectively, and by 73.2%, 67.1%, 65.9%, and 62.2% in comparison to CB, respectively. The lowest masses of shoots were observed after the application of Hp H (with a decrease of 30.7% and 25.6% compared to C and CB) and Ur L (with a decrease of 28.4% and 23.2%). The application of extract based on Ur L caused the highest root mass increase (126% and 165% higher mass than in C and CB), while the treatment with Vo R resulted in the lowest mass increase (7.4% lower mass than in C and 8.7% higher mass than in CB).

In general, the botanical extracts also increased the fresh mass of plants under stressful conditions (Table 2). For instance, the treatment with Ps S-derived products resulted in a mass increment of 44.8% and 35.5% under treatment with 100 mM concentration of sorbitol, 29.6% and 16.7% under 200 mM of sorbitol, and 19.2% and 14.8% under 300 mM of sorbitol. The least stimulating activity was noted for plants sprayed with Hp H—by 10.3% and 3.2% higher mass than in C and CB under 100 mM of sorbitol, respectively, and by 0% and 10% lower mass than in C and CB under 200 mM of sorbitol, respectively, and by 0% and 3.7% lower mass compared to C and CB under 300 mM of sorbitol, respectively. Under the lowest concentration of sorbitol, the extract based on Sg L increased the fresh mass of roots by 39.1% and 60.0% compared to C and CB, while the extract obtained from Tp F did not result in an increase in mass compared to C and increased by 15.0% compared to CB. In the mid-stressful conditions (exposure to 200 mM of sorbitol), the extract based on Hp H stimulated the mass growth to the greatest extent (by 22.7% and 58.8% compared to C and CB, respectively), while the extract derived from Tp F and So R exhibited the lowest activity (0% and 29.4% heavier than in C and CB, respectively). Under the highest concentration of sorbitol, the promoting activity of extracts was the lowest. In groups treated with the product obtained from Ps S, the masses of roots were the highest (by 23.1% and 23.1% than in C and CB, respectively), while plants sprayed with To F were the lightest (by 15.4% and 15.4% than in C and CB, respectively).

### 2.2. Concentration of Photosynthetic Pigments

In the majority of cases, the obtained extracts also improved the photosynthetic processes (Table 3). For example, the treatment with Ps S-based product increased the content of chlorophyll *a* by 51.4% and 43.2%, chlorophyll *b* by 10.3% and 23.1%, and carotenoids by 61.8% and 70.6% in comparison to C and CB, respectively. On the other hand, foliar spraying with Sg L had one of the lowest biostimulating activities. Its application increased the content of chlorophyll *a* (10.0% and 4.1% more than in C and CB, respectively) but decreased chlorophyll *b* (27.6% and 19.2% less than in C and CB, respectively) and carotenoids (4.5% less than in C and 0.7% more than in CB).

The foliar treatment with botanical extracts also demonstrated their beneficial properties during stressful conditions (Table 3). The product obtained from Ps S increased the content of chlorophyll *a* (by 40.6% and 38.5%), chlorophyll *b* (by 89.5% and 100%), and carotenoids (by 50.3% and 44.8%) to the highest extent in comparison to plants from C and CB groups grown under exposure to 100 mM of sorbitol, respectively. In groups treated with So R extract, the contents of chlorophyll *a* (4.7% and 3.1% higher than in C and CB, respectively), chlorophyll *b* (10.5% and 5.6% lower than in C and CB, respectively), and carotenoids (7.9% and 3.9% higher than in C and CB, respectively) were among the lowest in the experimental groups subjected to the lowest concentration of sorbitol. Under the increased stressful conditions (200 mM of sorbitol), for example, the extract based on Lc S showed the highest stimulating activity and enhanced the content of the aforementioned pigments by 37.5% and 30.5%, 47.4% and 40%, 32.4% and 25.8% in comparison to C and CB, respectively, while the extract based on To F exhibited the lowest activity, and despite that, it increased the content of chlorophyll *a* by 8.9% and 3.4% and decreased the content of chlorophyll *b* by 15.8% and 20.0% in comparison to C and CB, respectively, as well as increased the content of carotenoids by 1.7% when compared to C and decreased by 3.4% when compared to CB. In the conditions of the highest concentration of sorbitol, the extract obtained from Hp H enhanced the content of photosynthetic pigments by 69.0% and 42.0%, 114% and 100%, 34.7% and 30.9% in comparison to C and CB, respectively. The application of the extract based on Tp F increased the content of chlorophyll *a* by 4.8% compared to C but decreased by 12.0% compared to CB. The content of chlorophyll *b* was also lowered (by 14.3% and 20.0% compared to C and CB, respectively), while the content of carotenoids increased (by 0.5% more than in C) but decreased (by 2.4% less than in CB).

### 2.3. Concentration of Total Phenolic Compounds

Under conditions without stress, all tested botanical extracts increased the content of total phenolic compounds (TPC) in the model plant (Table 4). The highest amounts of TPC were indicated in seedlings treated with extracts obtained from Hp H (76.9% and 13.6% more than in C and CB, respectively), To L (62.8% and 4.2% more than in C and CB, respectively), and Ur L (59.0% and 2.1% more than in C and CB, respectively). On the contrary, the lowest values were reported in groups sprayed with extracts based on Lc S (1.2% more than in C and 35.0% less than in CB) and Vo R (4.7% more than in C and 32.8% more than in CB).

Plants from the experimental groups responded differently to the stressful conditions (Table 4). Under the lowest concentration of sorbitol (100 mM), none of the applied products increased the concentrations of total phenolic compounds. It can be seen that the amount of these compounds in the control group slightly increased (by 1.9%) in comparison to the conditions without sorbitol. The use of a commercial biostimulant enhanced their content by 3.9% compared to plants sprayed with water. Groups treated with Lc S (47.8% and 49.8% less than in C and CB, respectively) and Sg L (37.5% and 39.8% less than in C and CB, respectively) were characterised by the lowest concentrations of these compounds. Under the mid-stressful conditions (200 mM of sorbitol), the amount of TPC continued to decline in the control group (11.1% less than in C under 100 mM of sorbitol) as well as in the group treated with the commercial biostimulant (143% less than in C). However, the application of certain botanical extracts influenced the content of TPC—this can be observed in groups sprayed with Hp H-based extracts (48.5% and 261% more than in C and CB, respectively). The groups that had lower TPC content than groups treated with water were Ur L (14.6% less than in C and 108% more than in CB), Ps S (13.0% less than in C and 112% more than in CB), and Sg L (11.2% less than in C and 116% more than in CB). Under the most stressful conditions (300 mM of sorbitol), the content of TPC in the control group continued to decline (27.2% less than in C under 200 mM). Botanical extracts that stimulated the increase in the content of these compounds were To L (31.4% and 4.4% more than in C and CB, respectively) and Ur L (3.1% more than in C and 18.0% less than in CB).

### 2.4. Antioxidant Activities (DPPH, ABTS, and FRAP)

The antioxidant activity (Table 5), measured with the use of a DPPH assay, of seedlings grown under controlled laboratory conditions (without treatment with sorbitol) was the highest in groups treated with extracts derived from To L (44.6% and 32.7% more than in C and CB, respectively), Ps S (27.7% and 17.3% more than in C and CB, respectively), Vo R (15.8% and 6.4% more than in C and CB, respectively), Lc S (12.9% and 3.9% more than in C and CB, respectively), and So R (10.9% and 1.8% more than in C and CB, respectively). The differences between groups treated with the least stimulating extracts were not statistically significant. Under the least stressful conditions (100 mM of sorbitol), all tested botanical extracts increased the antioxidant activity, and those with the highest values included Lc S (276% and 203% more than in C and CB, respectively), Ps S (269% and 197% more than in C and CB, respectively), and Sg L (183% and 128% more than in C and CB, respectively). In most cases, similar observations can be seen under medium-stress conditions (200 mM of sorbitol). In the groups treated with botanical extracts, the antioxidant activity was higher than in the control group, and only four extracts (To F, Tp F, Sg L, and Lc S) decreased the antioxidant activity but in a statistically insignificant manner. The highest values were observed after the application of Ps S (59.5% and 67.5% more than in C and CB, respectively), To L (52.4% and 60.0% more than in C and CB, respectively), Ur L (44.0% and 51.3% more than in C and CB, respectively), and Hp H (42.9% and 50.0% more than in C and CB, respectively). However, the opposite trend can be noted in the case of the most stressful conditions, excluding the extract based on To L (2.7% and 1.3% more than in C and CB, respectively). The lowest antioxidant activity was found in the group sprayed with products obtained from Tp F (73.0% and 73.3% less than in C and CB, respectively) and Ur L (67.6% and 68.0% less than in C and CB, respectively).

The use of the ABTS assay (Table 5) showed that the antioxidant activity of plants grown under normal conditions (without treatment with sorbitol) was highest in groups sprayed with extracts derived from Ps S (77.6% and 287% more than in C and CB, respectively), Hp H (64.2% and 257% more than in C and CB, respectively), and To L (23.0% and 168% more than in C and CB, respectively), while it was lowest in groups treated with Sg L (74.1% and 43.6% less than in C and CB, respectively) and To F (65.0% and 23.8% less than in C and CB, respectively). Under exposure to 100 mM of sorbitol, the application of the extract obtained from To L was the only product that caused a decrease in the antioxidant activity (13.4% and 20.3% less than in C and CB, respectively), while other extracts such as Tp F (53.0% and 40.8% increase), So R (52.6% and 40.4% increase), and Vo R (48.4% and 36.6% increase) triggered its significant growth in comparison to C and CB, respectively. Plants exposed to both 200 mM and 300 mM concentrations of sorbitol and treated with botanical extracts showed greater antioxidant activity compared to plants sprayed with only water under the same conditions. The highest values of the tested factor were observed for Ps S (102% and 45.9% more than in C and CB, respectively) and Lc S (90.3% and 37.6% more than in C and CB, respectively) for groups subjected to 200 mM of sorbitol and for So R (213% more than in C and 19.3% less than in CB) and Tp F (187% more than in C and 26.0% less than in CB) for plants exposed to 300 mM of sorbitol.

The results of the FRAP assay (Table 5) showed that botanical extracts (with the exception of Vo R) can enhance the antioxidant activity of cabbage seedlings. For instance, the following extracts can be successfully applied on plants grown in stress-free conditions: To L (65.5% and 61.9% more than in C and CB, respectively), Hp H (37.1% and 34.2% more than in C and CB, respectively), Ps S (29.7% and 26.9% more than in C and CB, respectively), and Lc S (28.7% and 25.9% more than in C and CB, respectively). However, in low-stress conditions (100 mM of sorbitol), only So R significantly increased the antioxidant activity by 11.9% and 12.3% more than in C and CB, respectively. Other extracts, like Sg L, for example, decreased the measured parameter by 29.4% and 29.2% compared to C and CB, respectively. The opposite trend can be observed in plants subjected to medium stress. Most of the extracts increased the antioxidant activity, and those with the greatest stimulating activity included Vo R (71.8% and 98.1% more than in C and CB, respectively), Ur L (33.3% and 53.7% more than in C and CB, respectively), To L (25.2% and 44.4% more than in C and CB, respectively), and So R (24.7% and 43.7% more than in C and CB, respectively). In the case of the highest stress conditions (300 mM of sorbitol), the observed differences were mostly not statistically significant, with the exception of the application of Hp H (33.8% and 43.9%) and To F (12.9% and 21.5%), which increased the measured antioxidant activity compared to C and CB, respectively.

### 2.5. RNA-Seq Gene Expression

**Figure 1:**
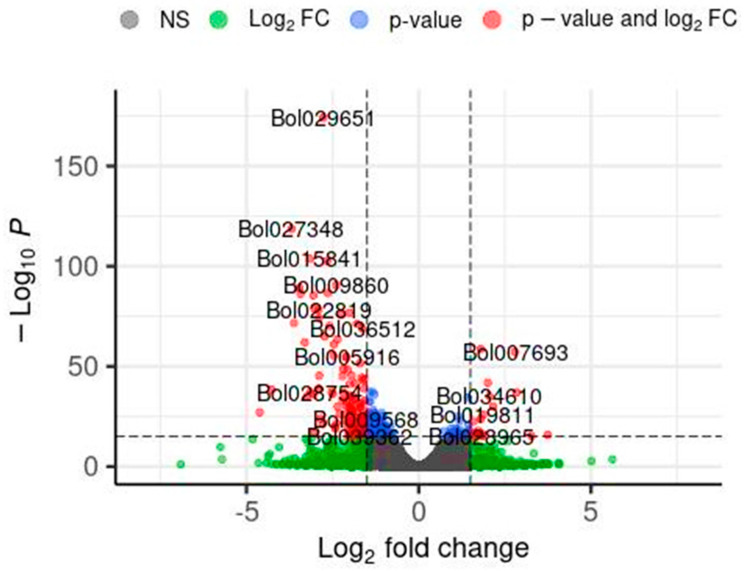
Volcano plot of differentially expressed genes (DEGs) identified by RNA-seq analysis. DEGs were selected with thresholds of *p* < 0.001 and log_2_FC > |1.5|. Each point in the differential expression volcano map represents a gene. Log_2_fold charge—logarithm of the differential expression of the gene in the samples. −Log_10_*P*—the negative logarithm of the statistically significant changes in gene expression. Grey—no significant DEG differences, Gein—above the log_2_FC but below the *p*-value thresholds, blue—above the *p*-value but below the log_2_FC thresholds, and red—DEGs meeting both significance thresholds.

In this section, the results of the impact of the application of the So R extract on the gene expression of cabbage seedlings under controlled laboratory conditions are presented. This extract was chosen as it demonstrated one of the most favourable effects on the tested parameters, including an increase in the length and mass of shoots of the model plant under both controlled and stressed conditions as well as the photosynthetic pigment content under the conditions of greatest stress. Differential gene expression analysis was performed using the DESeq2 software (ver. 1.30.0), and the results are shown in Figure 1. Volcano plots were used to display the diversity in gene expression levels between the control and induced treatments, which also indicated statistically significant differences. In total, 948 genes were differentially expressed, 245 were upregulated, and 703 were downregulated. The illustration shows selected genes that may have a potential impact on the regulation of plant development. Table 6 shows the detailed DEG results for the selected genes marked in Figure 1.

**Figure 2:**
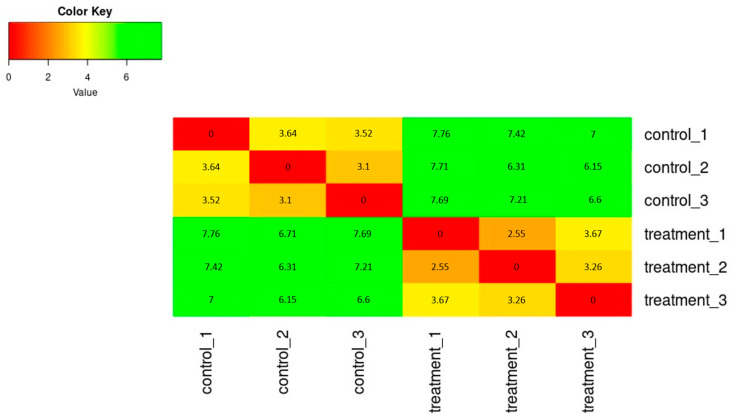
The heatmap of expression similarity between samples shows the distance between samples, calculating the SERE coefficient between each pair of samples.

**Figure 3:**
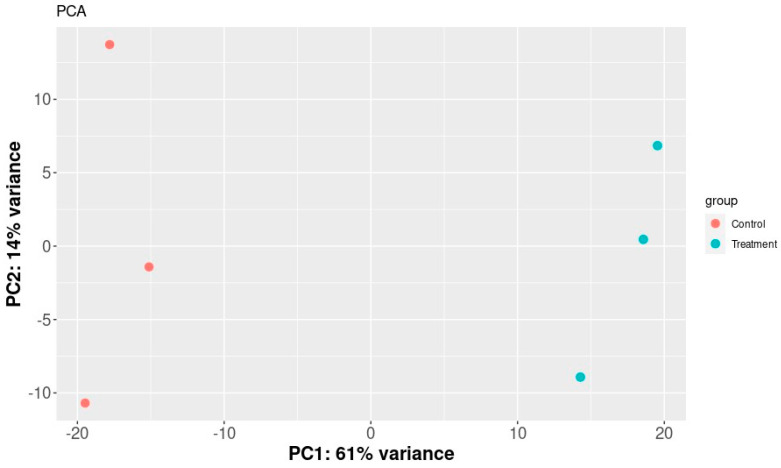
PCA plot of samples. It shows a scatter plot of the samples along the first two principal components.

The SERE (Simple Error Ratio Estimate) (Figure 2) and PCA (Principal Component Analysis) (Figure 3) plots show the clustering control group (sprayed with water) against samples treated with extracts. The plots indicate no significant impact on differentiation in gene expression caused by unintended experimental factors.

**Figure 4:**
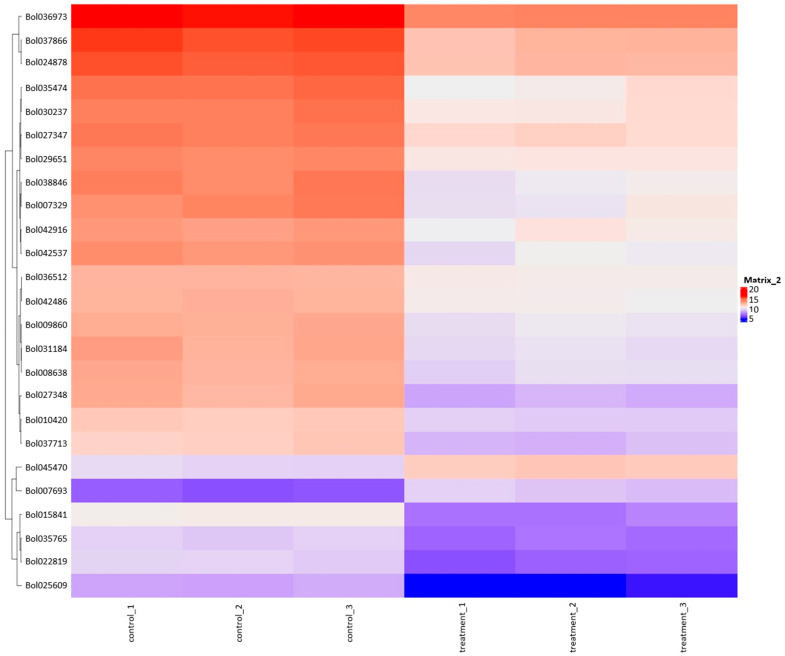
Heatmap plot combined with clustering group genes based on the similarity of their gene expression pattern.

The heatmap plot facilitates the identification of gene groups commonly regulated by signatures associated with treatment (Figure 4). Table 6 provides information about genes and the results of Differentially Expressed Genes (DEGs) for selected genes marked on this figure. The heatmap results reveal a substantial cluster of genes exhibiting downregulated expression in response to the plant extract treatment compared to control samples. The So R extract treatment reduced the expression of genes related to photosynthesis (chlorophyll A-B binding protein), stress response proteins (heat shock proteins, DnaJ Chaperone, FKBP-type peptidyl-prolyl cis-trans isomerase, glutathione S-transferase, Aldolase-type TIM barrel, and GDSL lipase/esterase) and some genes that may affect the regulation of gene expression (S-adenosylmethionine-dependent methyltransferases and pre-mRNA splicing Prp18-interacting factor).

**Figure 5:**
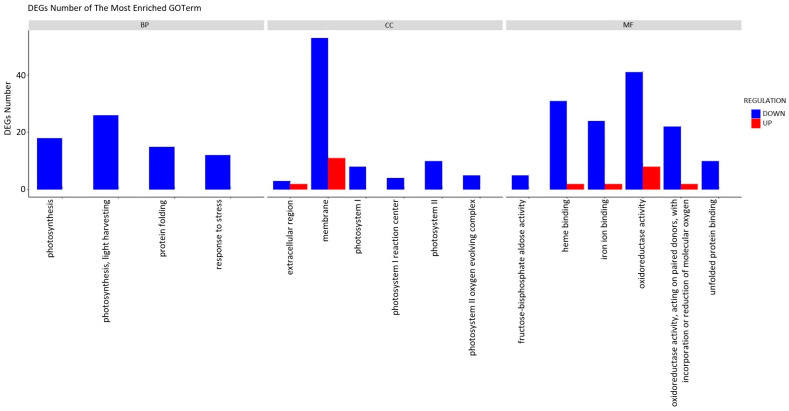
Functional annotation of differentially expressed genes (DEGs) using Gene Ontology (GO) classification analyses. The annotations are divided into three major functional categories: biological process (BP), cellular component (CC), and molecular function (MF).

To more accurately elucidate the impact of the extract treatment on the growth of cabbage seedlings, a Gene Ontology (GO) classification analysis was conducted (Figure 5). The results of the GO analysis indicated that the alterations in gene expression were primarily linked to the downregulation of specific functional processes. The plants subjected to induction exhibited a diminished performance in essential biological processes (BP), including photosynthesis, protein folding, and stress response, in comparison to the control group. When characterising the impact on cellular components (CC), a notable decrease in gene activity associated with membrane construction and photosynthetic systems becomes apparent. The impact of the treatment on photosynthesis was evident even at the molecular function (MF) level, with noticeable alterations in heme and iron ion binding, as well as oxidoreductase activity. The decrease in unfolded protein binding function activity is associated with the previously observed overall reduction in the activity of stress response genes.

## 3. Discussion

Accessible reports on the effects of biostimulant application to the highest extent indicate alterations in plant growth, development, and quality [14,24,25] and the amplification of crop stress tolerance [25,26], however, very often without addressing functional aspects [14]. Knowledge about the mechanism of action of most biostimulants is negligible or even unrecognised [14,25,27,28,29]. In most instances, the total composition of these products remains unidentified [27], and the analysis of individual bioactive compounds does not provide any direct correlation between their presence and the beneficial effects of biostimulant application that can be found in plants [21,28,29]. Moreover, the separation of the influence of one or more bioactive compounds from the effects of additional ingredients is usually highly complicated [27,30] and unfounded as their efficacy is presumably the result of the actions of several molecules [30]. This issue is also hampered by the presence of naturally occurring or commercially added amino acids, micronutrients, sugars, etc., which may exhibit synergistic, complementary, or no effects or may have been added for marketing or commercial registration purposes only [27]. For this reason, to obtain more stable bioproducts, it is of the utmost importance to attempt to reduce the heterogeneity of raw materials and to standardise protocols for their preparation and extraction [30]. Our previously published works focused on exploring the chemical composition of the botanical extracts [31,32]. The presence of the following bioactive compounds was confirmed: saponins (Ur L, Sg L, To F, To L, Tp F, Vo R, Hp H, So R, Ps S, and Lc S), oils and fats (Sg L, To F, To L, Tp F, Vo R, Hp H, and So R), alkaloids (Sg L, To L, Vo R, Hp H, So R, Ps S, and Lc S), triterpenes (Ur L and Tp F), terpenoids (Vo R), phytosterols (Sg L, To L, Hp H, and So R), steroids (Tp F and Ps S), phenolic compounds (Ur L, Sg L, To F, To L, Tp F, Vo R, Hp H, and So R), tannins (Ur L, Sg L, To F, To L, Tp F, Vo R, Hp H, So R, Ps S, and Lc S), anthocyanins (Ur L, Sg L, To F, Tp F, Hp H, and So R), coumarins (Ur L, Tp F, Hp H, Ps S, and Lc S), flavones (Ur L, Sg L, To F, Tp F, Hp H, So R, Ps S, and Lc S), flavonoids (Ur L, Sg L, To F, To L, Tp F, Vo R, Hp H, So R, Ps S, and Lc S), quinones (To L and So R), glycosides (Ur L, Sg L, Tp F, So R, Ps S, and Lc S), cardiac glycosides (Ur L, Sg L, To F, To L, Vo R, Hp H, and So R), proteins and amino acids (To F, To L, Tp F, Vo R, So R, Ps S, and Lc S), resin (To F, Vo R, So R, Ps S, and Lc S), sugars (Ur L, Sg L, To F, To L, Tp F, Vo R, Hp H, So R, Ps S, and Lc S), and vitamin C (Ps S and Lc S). In addition, the analyses of plant hormones were also provided.

This article is the beginning of a series of works aimed at clarifying the mechanisms of the action of extracts. In future articles, we will attempt to decipher the mechanism of their action under osmotic stress. This research has shown that the botanical extracts, obtained by means of ultrasound-assisted extraction, stimulated plant growth under controlled laboratory conditions, which was confirmed in Petri dish tests. To obtain the longest aboveground parts of crops, it is recommended to use bioproducts manufactured based on So R and Ur L, and to reduce the growth of unwanted plants, the Hp H extract could be considered (even at higher concentrations). For the cultivation of crops in which the largest root system is desired, we suggest the use of extracts obtained from To L, Tp F, and To F. The application of the Lc S-based extract should not significantly affect the development of the underground parts. The usage of botanical extracts can also provide positive results in increasing the fresh mass of cultivated plants. Extracts based on Lc S, Ps S, So R, and Ur L can stimulate the production of fresh mass by plants, while those based on Hp H and Ur L can contribute to its significant reduction (in the case of Hp H, the length of the shoots also decreased). However, the application of Ur L-based extracts caused the highest increase in root mass, while those based on Vo R caused the lowest increase in mass. Along with the improvement of plant growth, beneficial effects of the extracts on the content of photosynthetic pigments were also observed. For example, the application of extracts obtained from Ps S can increase the content of chlorophyll a, chlorophyll b, and carotenoids, while foliar spraying with Sg L showed one of the lowest stimulating activities of the photosynthetic process. To increase the content of total phenolic compounds, the application of Hp H, To L, and Ur L can be considered. The use of Lc S and Vo R did not exert statistically significant effects on plants. In general, regarding the increase in antioxidant activity, the extracts derived from To L, Ps S, Vo R, Lc S, and So R (DPPH assay); Ps S, Hp H, and To L (ABTS assay); To L, Hp H, Ps S, and Lc S can be successfully applied on plants grown in stress-free conditions. However, the use of Sg L and To F (ABTS assay) and Vo R (FRAP assay) may exhibit the least stimulating properties. The bioactivity and composition of biostimulants are not identical and depend on many factors, such as raw material type, location of raw material acquisition, extraction method, dose, concentration, application method, species and cultivar of crops, and environmental conditions [33]. For this reason, an attempt to chemically standardise bioproducts is of utmost importance. The presence of bioactive compounds, contributing to the beneficial effects of biostimulants, was evaluated in our previous works [31,32]. The performed analysis of botanical extracts indicated the presence of alkaloids, amino acids, carboxylic acids, elements, glycosides, hormones (abscisic acid, benzoic acid, gibberellic acid, indole acetic acid, jasmonic acid, salicylic acid, zeatin, zeatin riboside, and isipentenyl adenine), oils and fats, phenolic compounds (phenols, tannins, anthocyanins, coumarins, flavones, and flavonoids), proteins, quinines, quinones, resins, saponins, steroids, sugars, and vitamin C. Additionally, the antioxidant activity was identified. The observed positive effects of the obtained botanical extracts could be due to the activity of individual components or their synergistic action. Within the framework of this work and to strengthen our knowledge of the mechanism of action of botanical extracts, the transcriptomic analysis was conducted. These analyses allow the assessment of the alterations in the expression of genes; however, the special emphasis on the interplay of various phytohormones involved in signal transduction is necessitated [30]. The expression levels of known genes associated with the specific metabolic pathways induced by the bioproduct treatments can also be examined by qRT-PCR analysis. Sequencing techniques (NGS, especially RNA-seq) are increasingly being applied to explore mechanisms of action of biostimulants in light of the limitations of the microarray technique [30,34]. In addition, to clarify the unknown mechanisms of the activity of bioproducts, the RNA-seq approach could be a successful strategy for the development of innovative formulations [30].

Crops are frequently subjected to various types of adverse conditions (e.g., salinity and drought), which lead to reduced plant growth and productivity. The majority of these environmental constraints reduce the environmental water potential and cause plant osmotic stress. The consequences of osmotic stress manifest themselves in the inhibition of cell elongation, stomatal closure, reduction of photosynthetic activity, disruptions in water and ion uptake, translocation of assimilates, and changes in various metabolic processes [22]. Drought stress affects all plant growth parameters and response genes, as well as decreases membrane integrity and cell size, which leads to the formation of reactive oxygen species (including superoxide, hydroxyl, perhydroxy, and alkoxy radicals) and eventually cell death [35,36]. In order to combat the negative effects of environmental disturbances, plants develop comprehensive defence mechanisms, using physical adaptation and integrated molecular and cell reactions. The perception of abiotic stress activates the production of reactive oxygen species, an important secondary transmitter and early response mechanism. Under optimal growth conditions, ROS levels are at a moderate and balanced level due to the action of antioxidants and enzymes, making these molecules great signal transmitters. Nevertheless, abiotic stress leads to excessive ROS production that can lead to cell death. Reactive oxygen species include hydrogen peroxide (H_2_O_2_), hydrogen radical (OH), singlet oxygen (^1^O_2_), and superoxide anion (O_2_), which are formed in a certain pathway. Their accumulation allows plants to survive and adapt to various stressful factors [37]. Plants can also amplify the initial stress signals with phytohormones, such as ABA (abscisic acid). The phytohormone accumulation is connected to early plant stress signalling incidents (e.g., rapid ROS production). For example, the accumulation of ABA under a water deficit is contingent on ROS production via the NADPH oxidase. This ABA-induced ROS accumulation can enter guard cells and activate Ca^2+^ channels, which leads to an increase in the cytoplasmic Ca^2+^ concentration and, therefore, induce stomatal closure [37,38]. Studies show that biostimulants, due to their anti-stress nature, induce changes in the production of ROS and associated enzyme scavenging activities working to control oxidative damage [14,25], along with membrane stability, osmo-protection, and ion homeostasis [25]. The application of biostimulants evokes a wide array of changes in the abundance of mRNA transcripts, activating various biochemical pathways and physiological responses [30], which leads to a series of metabolic intracellular changes [25,30].

In recent years, new studies are beginning to change our understanding of the mechanisms of action of biostimulants. Despite this, it remains a major challenge to fully delineate their modes and mechanisms of action in plants. It is worth noting that the mode of action is the anatomical or functional alteration arising from the exposure to various factors (e.g., substances), while the mechanism of action specifies these changes at the molecular level [14]. Due to diverse mechanisms of action, within this discussion, we will focus on the action of one of the main categories of plant biostimulants—seaweed extracts and botanicals. However, attempts to elucidate the mechanism of action of biostimulants are mainly carried out for algal extracts, and such studies are lacking for plant extracts. Therefore, references to our literature will focus on the brown alga *Ascophyllum nodosum* (AN), which is the most widely used raw material for biostimulant production. In the literature, a growing number of reports on the mechanisms of action of its extracts (ANEs) appeared in recent years. One of the extensive reviews, provided by De Saeger et al. [39], showed that the application of ANEs in the cultivation of, among others, *Arabidopsis thaliana* (thale cress), *Brassica napus* (oilseed rape), *Cucumis sativus* (cucumber), *Daucus carota* (carrot), *Glycine max* (soybean), *Nicotiana tabacum* (tobacco), *Solanum lycopersicum* (tomato), and *Spinacia oleracea* (spinach), subjected to various abiotic (freezing, drought, and salinity) and biotic stresses, modify the expression of genes involved in the transportation of nutrients across the cell membrane, influence the endogenous balance of hormones, alleviate stress-related reactions, and enhance photosynthesis. They also drew attention to the fact that the wide variety of plant responses to bioproducts is stymied by many factors, such as (1) the non-unified experimental setups, (2) the different concentrations of ANE, (3) the incomplete information on composition, (4) the low replicability caused by the seasonal variations in algae compositions, (5) the occurrence of species-dependent effects, and (6) the complex composition of extracts [39]. In more detail, the use of ANEs in the cultivation of *A. thaliana* under drought stress reduced the expression of *NCED3* (*At3g14440*), involved in ABA biosynthesis, and *MYB60* (*At1g08810*), a transcription factor involved in stomatal regulation and increased the expression of the ABA-responsive genes *RAB18* (*At5g66400*) and *RD29A* (*At5g52310*). The authors also noted a decrease in gene expression of photosynthesis-related *RBCS1A* (*At1g67090*) and *RCA* (*At2g39730*) and of *PIP1;2* (*At2g45960*) and *βCA1* (*At3g01500*), which are involved in the regulation of CO_2_ diffusion in the mesophyll. Moreover, an increase in the expression of *PsbS* (*At1g44575*) and *VDE* (*At1g08550*) in the experimental groups indicated energy dissipation and improved *DRF* (*At5g42800*) and *SOD* (*At1g8830*) expression at the activation of the antioxidant defence system that prevented oxidative damage to PSII. The observed changes in the molecular pathways connected to enhanced drought tolerance after the application of ANE were revealed [40]. The currently available articles on the activity of *Ascophyllum nodosum*-based extracts showed a differential influence on *A. thaliana* transcriptome after the use of *Ascophyllum nodosum* extracts. The modification of the expression of genes involved in the gluconeogenesis/glyoxylate cycle, oxidative stress, and hormone metabolism was observed. An upregulation of the glutaredoxin family genes (*At1g0320* and *GRXC2*) and the cold-regulated gene *COR15A* was presented [41]. Another study on the activity of these brown algae products was conducted by Rasul et al. [42]. The authors noted a strong decrease in drought-induced damages in *Arabidopsis thaliana* induced by the regulation of key genes involved in the coordination of plant growth (*RD26* and *BES1*) and cell cycle (*CYCP2;1*). Elansary et al. [35] investigated the foliar application of extracts from *Ascophyllum nodosum* on *Paspalum vaginatum* (seashore paspalum) during prolonged irrigation intervals and under saline conditions. The authors observed significant increases in antioxidant activities, SOD (superoxide dismutase), CAT (catalase), and APX (ascorbate peroxidase) enzyme activities, leading to ROS (H_2_O_2_) depletion in plants treated with seaweed extract. The survey conducted by Shukla et al. [43] revealed that the application of the AN-based extract on *Glycine max* led to changes in the expression of stress-responsive *GmCYP707A1a* and *GmCYP707A3b* genes, involved in ABA catabolism. Moreover, the expression of the ABA-inducible *GmDREB1B* and the *BURP* domain protein-encoding *GmRD22* increased, while the expression of the ABA-independent stress-responsive gene *GmRD20* remained unchanged. Induced expression of the ABA-responsive fibrillin gene *FIB1a*, the aquaporin gene *GmPIP1b*, the glutathione S-transferase gene *GmGST*, the molecular chaperone *GmBIP*, and the antiquitin-like *GmTP55* was also observed in plants treated with ANE. Furthermore, extracts obtained from *Ascophyllum nodosum* modify the expression of *RbCS1A* and *RCA* genes (associated with the *RuBisCO* activation) [44], positively regulate the expression of *P5CS1* and *P5CS2*, genes linked to the biosynthesis of proline, negatively regulate the expression of other genes related to proline catabolism in *Arabidopsis* [45], and enhance the activity of antioxidant enzymes (e.g., CAT, SOD, and APX) and the production of antioxidants (e.g., ascorbate), leading to lower ROS accumulation and membrane damage in plants (e.g., *Phaseolus vulgaris*—common bean and *Paspalum vaginatum*) [35,46,47]. In the study by Ali et al. [48], the application of ANE in the cultivation of tomato and sweet pepper increased the upregulation of gene transcripts *Ga2Ox*, *IAA,* and *IPT* implicated in the biosynthesis of gibberellin, auxin, and cytokinin, respectively [48], while the treatment of oilseed rape caused an increase in the expression of *COPT2*, a gene coding for a Cu transporter, *BnSULTR1.1* and *BnSULTR1.2*, genes coding for sulfate transporters, and *BnNRT1.1* and *BnNRT2.1*, genes coding for nitrate transporters [49].

## 4. Materials and Methods

### 4.1. Chemicals

The following chemicals were used in this study: acetone (Stanlab, Lublin, Poland), calcium carbonate (Chempur, Piekary Śląskie, Poland), sodium carbonate (Sigma Aldrich, Poznań, Poland), ethanol (TH.GEYER, Warszawa, Poland), methanol (TH.GEYER), potassium persulphate (Sigma Aldrich), acetic acid (Supelco, Poznań, Poland), sodium acetate (Sigma Aldrich), Folin–Ciocalteu’s phenol reagent (Sigma Aldrich), Trolox (Sigma Aldrich), gallic acid (Sigma Aldrich), diphenyl-2-picrylhydrazyl (DPPH) (Sigma Aldrich), az-ino-bis-3-ethylbenzthiazoline6-sulphonic acid (ABTS) (Sigma Aldrich), and tri-pyridyl-S-triazine (TPTZ) (Sigma Aldrich). All reagents were of analytical grade. For the genetic analysis, the following were applied: Trizol Reagent (Life Technologies, Warszawa, Poland), a High-Capacity cDNA Reverse Transcription Kit (Applied Biosystems, Warszawa, Poland), a DyNAmo HS SYBR Green qPCR Kit (Thermo Fisher Scientific, Warszawa, Poland), and a MirVana™ miRNA Isolation Kit (Thermo Fisher Scientific).

### 4.2. Plant Materials

Based on our previous studies on the impact of potential biostimulants on model plants [50,51,52,53,54,55], we selected ten raw materials for further comprehensive analysis. In most cases, these raw materials are considered weeds and were chosen based on their prevalence in Europe. They were purchased from herbal stores. The dried biomasses were ground (500 μm) and appropriately stored in stringed bags at room temperature without access to light. The biomasses of Ur L (*Urtica dioica* L.; leaves), Sg L (*Solidago gigantea* Aiton; leaves), To F (*Taraxacum officinale* F.H. Wiggers coll.; flowers), To L (*Taraxacum officinale* F.H. Wiggers coll.; leaves), Tp F (*Trifolium pratense* L.; flowers), Vo R (*Valeriana officinalis* L.; roots), Hp H (*Hypericum perforatum* L.; herb), So R (*Symphytum officinale* L.; roots), Ps S (*Pisum sativum* L.; seeds), and Lc S (*Lens culinaris* Medik.; seeds) were purified, dried (50 °C), ground (500 μm), averaged, and stored in string bags without access to light.

### 4.3. Extract Production

Extracts tested on plants were selected based on our preliminary studies [50,51,52,53,54,55]. The chemical composition of analysed extracts is presented in our previous articles [31,32].

Extracts were produced by ultrasound-assisted extraction (UAE). The appropriately prepared raw materials were subjected to extraction with deionised water (1:20)—the mixtures were macerated (30 min, 23 ± 2 °C), exposed to ultrasounds (30 min, 40 W, homogeniser UP 50H, Hielscher Ultrasonics GmbH, Teltow, Germany), and then centrifuged (10 min, 4500 rpm, Heraeus Megafuge 40, rotor TX-750, Thermo Scientific, Waltham, MA, USA). The collected supernatants were used to prepare 0.5% extracts in deionised water.

### 4.4. Model Plant and Growth Conditions

The white head cabbage seedlings (“Sokrates”, Syngenta, Warszawa, Poland) were grown in a thermostatic cabinet (ST 1450 CS SMART, POL-EKO sp.k., Wodzisław Śląski, Poland) (24 °C day, 20 °C night) with a photoperiod (16 h day, 8 h night) under controlled laboratory (constant moisture level) and stress conditions (100, 200, and 300 mM of sorbitol) [56] for fourteen days. Fully randomised screening tests were carried out in Petri dishes (36 seeds per plate, with 10 repetitions). The foliar spray application of 0.5% extracts (1 mL) was performed on the sixth, eighth, and tenth days after sowing. Groups subjected to stress were watered with appropriate concentrations of sorbitol on the sixth (2 mL) and seventh days (3 mL). The control groups were treated with deionised water and a commercial biostimulant on the same days as the experimental groups. The selection of the concentration of extracts used in cabbage tests was made based on earlier research [50,51].

### 4.5. Biometric Measurements

Plants were collected and subjected to measurements of length of aboveground as well as root parts. The fresh masses were also assessed. For further chemical analysis, hypocotyl with cotyledons (“shoots”) were used.

### 4.6. Concentration of Photosynthetic Pigments

For the evaluation of the total chlorophyll (*a* + *b*) and carotenoid concentrations, the fresh cotyledons (0.2 g) were ground in a mortar with a few drops of 80% acetone and a pinch of sand and calcium carbonate. The obtained pastes were filtered and quantitatively transferred to the measuring flasks (50 mL). The absorbances (663, 645, and 470 nm) were immediately measured (3 replicates; UV-Vis spectrophotometer, HACH DR1900) [50].

### 4.7. Concentration of Total Phenolic Compounds

For the assessment of the total phenolic compounds (TPC) concentrations, the Folin–Ciocalteu method was chosen. The fresh and homogenous aboveground parts of plants (2 g FM) were mixed with methanol (80%, 20 mL), sonicated (15 min), and centrifuged (10 min, 4500 rpm). The solutions containing supernatants (0.1 mL), Folin–Ciocalteu’s phenol reagent (0.2 mL), and distilled water (2.0 mL) were incubated (3 min, 23 ± 2 °C) and then mixed with sodium carbonate (20%, 1.0 mL), and left to react (1 h, no access to light). The absorbance (765 nm) was measured (HACH DR1900 spectrophotometer, Berlin, Germany), and the results were expressed as gallic acid equivalents (GAE) (mg·100 g^−1^ FM).

### 4.8. Antioxidant Activities (DPPH, ABTS, and FRAP)

The experimental protocols for the assessment of antioxidant activities were carried out according to our previous works [50,52]. For the measurements of the DPPH assay, supernatants (0.5 mL, ten folded with 80% methanol), ethanol (1.5 mL), and DPPH solution (0.5 mL) were mixed and left to react (10 min, 23 ± 2 °C, in the dark). The absorbance was measured at 517 nm. For the ABTS assay, the supernatants (30 μL, ten-folded with 80% methanol) and ABTS solution (3 mL) were mixed and left to rest for 6 min under dark conditions. The absorbance was read at 734 nm. For the FRAP assay, the supernatants (1 mL) and FRAP reagent (3 mL) were allowed to react (10 min). The absorbance at 593 nm was determined. The results were expressed in µM Trolox·g^−1^ FM.

### 4.9. RNA Preparation Prior to Sequencing

RNA was extracted from cabbage seedling tissue using the mirVana™ miRNA Isolation Kit (Thermo Fisher) following the manufacturer’s instructions. DNase I treatment (Thermo Fisher) was performed to remove genomic DNA. RNA quality was verified by BGI Genomics. RNA sequencing was performed using BGI technology, employing a 150 bp paired-end (PE) approach. All sequenced samples obtained yielded below 20 million raw reads (>7 Gbp) with a quality index Q30 below 90%.

### 4.10. Statistical and Bioinformatical Data Analysis

For the statistical analysis of the biometric measurements and the analysis of chemical compounds and antioxidant activity assays, the STATISTICA software ver. 13.3 (TIBCO Software Inc., Tulsa, OK, USA) was used. To verify the normality of the data, the Shapiro–Wilk test was applied. For the normal distribution, to evaluate the homogeneity of variance the Brown–Forsythe test was used. Differences were evaluated with the Tukey’s Honest Significant Difference (HSD) test (significant difference when *p* < 0.05). The Kruskal–Wallis test was employed for data without normal distribution. Statistically significant differences between the examined extracts and the control group (C) were marked with “*a*”, while the differences between the extracts and the commercial product (CB) were marked with “*b*”.

Raw sequencing reads were subjected to quality control (QC) using FastQC [57] and subsequently trimmed for quality and adapter sequences using Trimmomatic [58].

Read mapping and processing: The high-quality reads were then mapped to the *Brassica oleracea* reference genome, which was obtained from the RefSeq database, using Bowtie2 [59]. The alignment generated BAM files, which were sorted and indexed for downstream analysis. The library preparation was strand-specific, and accordingly, all analyses were performed using strand-specific options to maintain the orientation integrity.

Count matrix and differential expression analysis: FeatureCounts [60] was utilised to create a count matrix from the sorted BAM files. Differential expression analysis was performed using DESeq2 [61], which identifies differentially expressed genes under various conditions.

Gene Ontology and enrichment analysis: For the Gene Ontology (GO) enrichment analysis, we utilised the Bioconductor package goseq in R. This package is specifically designed for GO enrichment analysis in RNA-seq datasets, taking into account the length bias in gene selection inherent to this type of data [62]. The goseq analysis enabled us to identify significant GO terms associated with differentially expressed genes, thereby providing insights into biological processes, molecular functions, and cellular components that are potentially implicated in our study of *Brassica oleracea*.

SERE coefficient and heatmap visualisation: The SERE coefficient, a measure of expression regulation, was calculated for each sample. The results were visualised as a heatmap, providing a comprehensive overview of expression regulation across samples.

Statistical analysis and data visualisation: All statistical analyses and data visualisations were conducted in R, utilising the package ggplot2 (ver. 3.4) [63]. This comprehensive approach ensured a thorough exploration and presentation of the RNA-seq data.

## 5. Conclusions

This is the first study investigating the mechanisms driving the positive effects of novel botanical extracts applied to cabbage seedlings grown under controlled laboratory conditions. It was observed that under drought conditions (induced by the addition of sorbitol), the treatment with biostimulants significantly improved the performance of the model plant, indicating an increase in plant tolerance to adverse growth conditions. Foliar spraying with To L (under 100 mM of sorbitol), Tp F (under 200 mM of sorbitol), and To F (under 300 mM) extracts stimulated the length of the shoots in comparison to the control group treated with water. The least stimulating activity was exhibited by Hp H extracts. In the case of roots, the use of the extract based on Sg L increased their length to the highest extent, while the Ur L-based extract was the only product that significantly reduced the root length (only under 300 mM of sorbitol). In general, the usage of novel botanical extracts can also enhance the fresh mass of shoots and roots of plants. For example, the treatment with Ps S-derived product resulted in favourable effects on the aboveground biomass in low- as well as high-stress conditions, while Hp H extracts exhibited the least stimulating activity. The use of Sg L (under 100 mM of sorbitol), Hp H (200 mM of sorbitol), and Ps S (300 mM of sorbitol) stimulated root mass growth to the greatest extent, while the application of Tp F (100 mM), Tp F, So R (200 mM of sorbitol), and To F (300 mM of sorbitol) showed the lowest activity. Treatment with Ps S (under 100 mM of sorbitol), Lc S (under 200 mM of sorbitol), and Hp H (under 300 mM of sorbitol) increased the photosynthetic process in plants, while the application of So R (under 100 mM of sorbitol), To F (under 200 mM of sorbitol), and Tp F (under 300 mM of sorbitol) exhibited the lowest activity in the enhancement of photosynthesis. Plants treated with botanical extracts responded differently to the stressful conditions. Under the lowest stressful conditions (100 mM of sorbitol), none of the obtained bioproducts increased the concentrations of total phenolic compounds. Under the mid-stressful conditions (200 mM of sorbitol), the application of Hp H stimulated the content of TPC to the highest extent, while the use of Ur L, Ps S, and Sg L exhibited the lowest activity. Under the highest stressful conditions (300 mM of sorbitol), To L and Ur L stimulated an increase in the content of these compounds. As shown by the DPPH assay, the use of all extracts under 100 mM of sorbitol can increase the antioxidant activity, and those with the highest values included Lc S, Ps S, and Sg L. Similar observations can be made for 200 mM of sorbitol in the majority of experimental groups for plants treated with Ps S, To L, Ur L, and Hp H. Only four extracts (To F, Tp F, Sg L, and Lc S) decreased antioxidant activity. The opposite trend can be noted for 300 mM of sorbitol, excluding the extract based on To L. The lowest antioxidant activity was found in the group sprayed with products obtained from Tp F. The highest antioxidant activity, measured with the use of the ABTS assay, under exposure to 100 mM of sorbitol, was observed after the application of the extract obtained from Tp F, So R, and Vo R, while the extract based on To L was the only product that decreased antioxidant activity. Plants exposed to both 200 mM and 300 mM concentrations of sorbitol and treated with botanical extracts (e.g., with Ps S and Lc S under medium stress and with So R and Tp F under the highest stress) showed greater antioxidant activity compared to plants sprayed with only water under the same conditions. However, under the low-stress conditions (100 mM of sorbitol), the antioxidant activity measured with the FRAP assay was higher only in the group treated with So R, while for other bioproducts (e.g., Sg L), it was lower. In mid-stress conditions (200 mM of sorbitol), most of the extracts increased the antioxidant activity (e.g., Vo R, Ur L, To L, and So R), while under the highest stress (300 mM of sorbitol) the observed differences were mostly not statistically significant, with the exception of the application of Hp H and To F. Additionally, our findings were confirmed by transcriptome studies, which showed that the application of a selected extract (So R) modified the expression of the following genes: *Bol029651* (glutathione S-transferase), *Bol027348* (chlorophyll A-B binding protein), *Bol015841* (S-adenosylmethionine-dependent methyltransferases), *Bol009860* (chlorophyll A-B binding protein), *Bol022819* (GDSL lipase/esterase), *Bol036512* (heat shock protein 70 family), *Bol005916* (DnaJ Chaperone), *Bol028754* (pre-mRNA splicing Prp18-interacting factor), *Bol009568* (heat shock protein Hsp90 family), *Bol039362* (gibberellin regulated protein), *Bol007693* (B-box-type zinc finger), *Bol034610* (RmlC-like cupin domain superfamily), *Bol019811* (myb_SHAQKYF: myb-like DNA-binding domain, SHAQKYF class), and *Bol028965* (DA1-like Protein). The GO functional analysis indicated that the application of extracts leads to a decrease in the expression of many genes related to the response to stress and photosynthetic systems, which may confirm a reduction in the level of oxidative stress in plants treated with biostimulants. Based on the current study, we are convinced that botanical extracts can help crops alleviate the side effects of prolonged drought periods observed in most agricultural areas of the world.

## Figures and Tables

**Table 1:** The length of shoots and roots of seedlings (cm, N = 50, mean ± SD).

Group	Without Stress		100 mM of Sorbitol		200 mM of Sorbitol		300 mM of Sorbitol	
	Shoots	Roots	Shoots	Roots	Shoots	Roots	Shoots	Roots
**C**	1.59 ± 0.06	6.02 ± 0.25	1.41 ± 0.05	5.64 ± 0.27	1.39 ± 0.07	4.88 ± 0.33	1.12 ± 0.04 *b*	4.02 ± 0.14 *b*
**CB**	1.66 ± 0.12	6.00 ± 0.10	1.53 ± 0.07	5.85 ± 0.31	1.47 ± 0.04	4.90 ± 0.18	1.44 ± 0.11 *a*	4.78 ± 0.32 *a*
**Ur L**	1.90 ± 0.09 *a*	6.24 ± 0.34	1.64 ± 0.11	5.89 ± 0.28	1.47 ± 0.08	5.34 ± 0.37	1.22 ± 0.06 *b*	3.02 ± 0.18 *a*,*b*
**Sg L**	1.84 ± 0.10	6.85 ± 0.52	1.71 ± 0.07 *a*	6.75 ± 0.49 *a*,*b*	1.32 ± 0.11	5.86 ± 0.33 *a*,*b*	1.14 ± 0.04 *b*	5.78 ± 0.32 *a*,*b*
**To F**	1.74 ± 0.09	7.45 ± 0.50 *a*,*b*	1.64 ± 0.11	6.08 ± 0.20	1.36 ± 0.10	5.61 ± 0.29 *a*	1.36 ± 0.03 *a*	4.15 ± 0.23
**To L**	1.84 ± 0.11	7.17 ± 0.50 *a*,*b*	1.75 ± 0.09 *a*	6.20 ± 0.41	1.57 ± 0.10	5.96 ± 0.31 *a*,*b*	1.19 ± 0.07 *b*	5.10 ± 0.28 *a*
**Tp F**	1.81 ± 0.06	7.29 ± 0.48 *a*,*b*	1.62 ± 0.12	5.58 ± 0.37	1.62 ± 0.04 *a*	5.47 ± 0.24	1.20 ± 0.08 *b*	5.45 ± 0.38 *a*
**Vo R**	1.80 ± 0.13	6.76 ± 0.18	1.74 ± 0.14 *a*	5.95 ± 0.32	1.52 ± 0.06	5.73 ± 0.30 *a*,*b*	1.22 ± 0.07 *b*	5.59 ± 0.36 *a*,*b*
**Hp H**	1.42 ± 0.12	7.13 ± 0.47 *a*,*b*	1.18 ± 0.04 *b*	5.94 ± 0.27	1.17 ± 0.05 *a*,*b*	5.36 ± 0.33	1.14 ± 0.05 *b*	4.19 ± 0.22
**So R**	1.94 ± 0.13 *a*,*b*	6.23 ± 0.41	1.71 ± 0.13 *a*	5.90 ± 0.30	1.57 ± 0.08	5.25 ± 0.25	1.22 ± 0.09 *b*	4.70 ± 0.25 *a*
**Ps S**	1.84 ± 0.12	6.88 ± 0.34	1.56 ± 0.09	5.74 ± 0.18	1.50 ± 0.10	5.53 ± 0.35	1.25 ± 0.06 *b*	4.43 ± 0.29
**Lc S**	1.87 ± 0.10 *a*	6.06 ± 0.42	1.55 ± 0.08	6.07 ± 0.30	1.53 ± 0.06	4.84 ± 0.27	1.28 ± 0.06 *b*	4.61 ± 0.31

*a*: Statistically significant differences (*p* < 0.05) between the control group (C) and extracts. *b*: Statistically significant differences (*p* < 0.05) between the commercial biostimulant (CB) and extracts. Abbreviations: Ur L—*Urtica dioica* L. (leaves), Sg L—*Solidago gigantea* Aiton (leaves), To F—*Taraxacum officinale* F.H. Wiggers coll. (flowers), To L—*Taraxacum officinale* F.H. Wiggers coll. (leaves), Tp F—*Trifolium pratense* L. (flowers), Vo R—*Valeriana officinalis* L. (roots), Hp H—*Hypericum perforatum* L. (herb), So R—*Symphytum officinale* L. (roots), Ps S—*Pisum sativum* L. (seeds), and Lc S—*Lens culinaris* Medik. (seeds). Comparisons were made within each column.

**Table 2:** The fresh mass of shoots and roots of seedlings (g, N = 5, mean ± SD).

Group	Without Stress		100 mM of Sorbitol		200 mM of Sorbitol		300 mM of Sorbitol	
	Shoots	Roots	Shoots	Roots	Shoots	Roots	Shoots	Roots
**C**	0.88 ± 0.06	0.27 ± 0.02	0.29 ± 0.01	0.23 ± 0.02	0.27 ± 0.01	0.22 ± 0.01 *b*	0.26 ± 0.01	0.13 ± 0.01
**CB**	0.82 ± 0.06	0.23 ± 0.02	0.31 ± 0.02	0.20 ± 0.02	0.30 ± 0.02	0.17 ± 0.01 *a*	0.27 ± 0.01	0.13 ± 0.01
**Ur L**	1.33 ± 0.10 *a*,*b*	0.61 ± 0.05 *a*,*b*	0.34 ± 0.02	0.24 ± 0.02	0.31 ± 0.02	0.23 ± 0.02 *b*	0.28 ± 0.02	0.14 ± 0.01
**Sg L**	0.63 ± 0.04 *a*,*b*	0.35 ± 0.03 *a*,*b*	0.35 ± 0.02 *a*	0.32 ± 0.03 *a*,*b*	0.33 ± 0.03 *a*	0.24 ± 0.02 *b*	0.31 ± 0.03 *a*	0.13 ± 0.01
**To F**	1.19 ± 0.06 *a*,*b*	0.30 ± 0.03 *b*	0.36 ± 0.03 *a*	0.25 ± 0.02	0.30 ± 0.02	0.23 ± 0.02 *b*	0.29 ± 0.03	0.11 ± 0.01
**To L**	1.12 ± 0.06 *a*,*b*	0.30 ± 0.03 *b*	0.35 ± 0.02 *a*	0.29 ± 0.03 *a*,*b*	0.33 ± 0.02 *a*	0.23 ± 0.02 *b*	0.27 ± 0.02	0.12 ± 0.01
**Tp F**	0.74 ± 0.04	0.32 ± 0.02 *b*	0.33 ± 0.02	0.23 ± 0.02	0.31 ± 0.02	0.22 ± 0.01 *b*	0.30 ± 0.02	0.13 ± 0.01
**Vo R**	0.82 ± 0.03	0.25 ± 0.02	0.36 ± 0.02 *a*,*b*	0.24 ± 0.02	0.32 ± 0.02 *a*	0.24 ± 0.02 *b*	0.26 ± 0.01	0.12 ± 0.01
**Hp H**	0.61 ± 0.04 *a*,*b*	0.30 ± 0.03 *b*	0.32 ± 0.03	0.30 ± 0.02 *a*,*b*	0.27 ± 0.02	0.27 ± 0.02 *a*,*b*	0.26 ± 0.02	0.12 ± 0.01
**So R**	1.36 ± 0.12 *a*,*b*	0.30 ± 0.01 *b*	0.38 ± 0.02 *a*,*b*	0.24 ± 0.02	0.32 ± 0.02 *a*	0.22 ± 0.01 *b*	0.32 ± 0.03 *a*,*b*	0.14 ± 0.00
**Ps S**	1.37 ± 0.08 *a*,*b*	0.31 ± 0.03 *b*	0.42 ± 0.03 *a*,*b*	0.27 ± 0.03 *b*	0.35 ± 0.01 *a*,*b*	0.26 ± 0.02 *b*	0.31 ± 0.02 *a*	0.16 ± 0.01 *a*,*b*
**Lc S**	1.42 ± 0.13 *a*,*b*	0.37 ± 0.03 *a*,*b*	0.36 ± 0.03 *a*	0.28 ± 0.02 *a*,*b*	0.36 ± 0.03 *a*,*b*	0.23 ± 0.02 *b*	0.29 ± 0.02	0.12 ± 0.00

*a*: Statistically significant differences (*p* < 0.05) between the control group (C) and extracts. *b*: Statistically significant differences (*p* < 0.05) between the commercial biostimulant (CB) and extracts. Abbreviations: Ur L—*Urtica dioica* L. (leaves), Sg L—*Solidago gigantea* Aiton (leaves), To F—*Taraxacum officinale* F.H. Wiggers coll. (flowers), To L—*Taraxacum officinale* F.H. Wiggers coll. (leaves), Tp F—*Trifolium pratense* L. (flowers), Vo R—*Valeriana officinalis* L. (roots), Hp H—*Hypericum perforatum* L. (herb), So R—*Symphytum officinale* L. (roots), Ps S—*Pisum sativum* L. (seeds), and Lc S—*Lens culinaris* Medik. (seeds). Comparisons were made within each column.

**Table 3:** The concentrations of chlorophyll *a* (mg·100 g^−1^ FM), chlorophyll *b* (mg·100 g^−1^ FM), and carotenoids (µg·100 g^−1^ FM) extracted from seedling cotyledons (N = 4, mean ± SD).

	Without Stress			100 mM of Sorbitol			200 mM of Sorbitol			300 mM of Sorbitol		
Group	Chlorophyll *a*	Chlorophyll *b*	Carotenoids	Chlorophyll *a*	Chlorophyll *b*	Carotenoids	Chlorophyll *a*	Chlorophyll *b*	Carotenoids	Chlorophyll *a*	Chlorophyll *b*	Carotenoids
**C**	0.70 ± 0.01 *b*	0.29 ± 0.01	21.86 ± 1.27	0.64 ± 0.00	0.19 ± 0.02	17.18 ± 0.81	0.56 ± 0.00 *b*	0.19 ± 0.01	16.81 ± 1.08	0.42 ± 0.00 *b*	0.14 ± 0.01	16.72 ± 0.78
**CB**	0.74 ± 0.01 *a*	0.26 ± 0.01	20.74 ± 1.08	0.65 ± 0.00	0.18 ± 0.01	17.84 ± 0.90	0.59 ± 0.00 *a*	0.20 ± 0.00	17.70 ± 1.11	0.50 ± 0.00 *a*	0.15 ± 0.01	17.21 ± 0.91
**Ur L**	0.87 ± 0.01 *a*,*b*	0.33 ± 0.01 *b*	23.24 ± 1.16	0.80 ± 0.00 *a*,*b*	0.22 ± 0.00 *b*	20.63 ± 1.53 *a*	0.74 ± 0.01 *a*,*b*	0.28 ± 0.02 *a*,*b*	22.48 ± 1.61 *a*,*b*	0.45 ± 0.01 *a*,*b*	0.12 ± 0.02 *b*	15.51 ± 0.77
**Sg L**	0.77 ± 0.01 *a*,*b*	0.21 ± 0.01 *a*,*b*	20.88 ± 1.16	0.75 ± 0.01 *a*,*b*	0.27 ± 0.01 *a*,*b*	20.53 ± 1.03	0.71 ± 0.01 *a*,*b*	0.25 ± 0.02 *a*	20.95 ± 1.19 *a*	0.56 ± 0.01 *a*,*b*	0.17 ± 0.01	16.96 ± 1.04
**To F**	0.88 ± 0.01 *a*,*b*	0.34 ± 0.02 *a*,*b*	26.44 ± 2.03 *a*,*b*	0.76 ± 0.00 *a*,*b*	0.30 ± 0.01 *a*,*b*	20.99 ± 1.12 *a*	0.61 ± 0.01 *a*	0.16 ± 0.01	17.10 ± 0.52	0.51 ± 0.01 *a*	0.14 ± 0.01	17.71 ± 0.80
**To L**	0.90 ± 0.00 *a*,*b*	0.24 ± 0.00 *a*	24.52 ± 1.64	0.69 ± 0.00 *a*,*b*	0.28 ± 0.01 *a*,*b*	19.09 ± 1.08	0.67 ± 0.01 *a*,*b*	0.29 ± 0.01 *a*,*b*	20.64 ± 1.04 *a*	0.47 ± 0.01 *a*,*b*	0.13 ± 0.01	16.93 ± 0.93
**Tp F**	0.88 ± 0.01 *a*,*b*	0.33 ± 0.01 *b*	25.70 ± 1.87 *b*	0.71 ± 0.00 *a*,*b*	0.27 ± 0.00 *a*,*b*	22.98 ± 1.54 *a*,*b*	0.63 ± 0.01 *a*,*b*	0.22 ± 0.06	17.00 ± 1.19	0.44 ± 0.01 *a*,*b*	0.12 ± 0.02 *b*	16.80 ± 0.86
**Vo R**	0.74 ± 0.01 *a*	0.29 ± 0.00	20.47 ± 1.59	0.67 ± 0.01 *a*	0.19 ± 0.01	22.93 ± 1.09 *a*,*b*	0.65 ± 0.02 *a*,*b*	0.15 ± 0.01 *b*	18.86 ± 0.75	0.64 ± 0.01 *a*,*b*	0.27 ± 0.01 *a*,*b*	18.92 ± 0.91
**Hp H**	0.86 ± 0.02 *a*,*b*	0.35 ± 0.04 *a*,*b*	27.30 ± 1.57 *a*,*b*	0.81 ± 0.00 *a*,*b*	0.26 ± 0.01 *a*,*b*	24.49 ± 1.67 *a*,*b*	0.72 ± 0.01 *a*,*b*	0.15 ± 0.01 *b*	20.98 ± 1.40 *a*,*b*	0.71 ± 0.00 *a*,*b*	0.30 ± 0.01 *a*,*b*	22.52 ± 0.25 *a*,*b*
**So R**	0.75 ± 0.00 *a*	0.29 ± 0.01	21.64 ± 1.01	0.67 ± 0.00	0.17 ± 0.02	18.54 ± 1.32	0.64 ± 0.01 *a*,*b*	0.24 ± 0.02	19.89 ± 0.94	0.63 ± 0.00 *a*,*b*	0.20 ± 0.01 *a*,*b*	20.29 ± 1.31 *a*,*b*
**Ps S**	1.06 ± 0.01 *a*,*b*	0.32 ± 0.01 *b*	35.38 ± 1.97 *a*,*b*	0.90 ± 0.02 *a*,*b*	0.36 ± 0.01 *a*,*b*	25.83 ± 1.16 *a*,*b*	0.83 ± 0.01 *a*,*b*	0.18 ± 0.00	20.38 ± 1.32 *a*	0.70 ± 0.00 *a*,*b*	0.26 ± 0.00 *a*,*b*	20.95 ± 1.04 *a*,*b*
**Lc S**	0.82 ± 0.00 *a*,*b*	0.17 ± 0.01 *a*,*b*	20.92 ± 1.23	0.79 ± 0.01 *a*,*b*	0.34 ± 0.01 *a*,*b*	23.77 ± 1.08 *a*,*b*	0.77 ± 0.00 *a*,*b*	0.28 ± 0.01 *a*,*b*	22.26 ± 1.69 *a*,*b*	0.53 ± 0.01 *a*,*b*	0.16 ± 0.01	17.19 ± 1.02

*a*: Statistically significant differences (*p* < 0.05) between the control group (C) and extracts. *b*: Statistically significant differences (*p* < 0.05) between the commercial biostimulant (CB) and extracts. Abbreviations: Ur L—*Urtica dioica* L. (leaves), Sg L—*Solidago gigantea* Aiton (leaves), To F—*Taraxacum officinale* F.H. Wiggers coll. (flowers), To L—*Taraxacum officinale* F.H. Wiggers coll. (leaves), Tp F—*Trifolium pratense* L. (flowers), Vo R—*Valeriana officinalis* L. (roots), Hp H—*Hypericum perforatum* L. (herb), So R—*Symphytum officinale* L. (roots), Ps S—*Pisum sativum* L. (seeds), and Lc S—*Lens culinaris* Medik. (seeds). Comparisons were made within each column.

**Table 4:** The total phenolic content (mg of gallic acid equivalents (GAE)·100 g^−1^ FW) of seedlings (N = 4, mean ± SD).

Group	Without Stress	100 mM of Sorbitol	200 mM of Sorbitol	300 mM of Sorbitol
**C**	61.06 ± 4.73 *b*	62.24 ± 4.02	56.02 ± 3.73 *b*	40.81 ± 1.90 *b*
**CB**	95.10 ± 7.26 *a*	64.68 ± 2.32	23.04 ± 1.77 *a*	51.34 ± 2.44 *a*
**Ur L**	97.09 ± 5.82 *a*	54.79 ± 2.83 *b*	47.82 ± 3.86 *b*	42.09 ± 3.37 *b*
**Sg L**	80.16 ± 3.60 *a*	38.91 ± 1.56 *a*,*b*	49.75 ± 4.74 *b*	39.72 ± 3.50 *b*
**To F**	76.67 ± 5.46 *b*	44.85 ± 3.24 *a*,*b*	57.38 ± 3.64 *b*	35.95 ± 2.30 *b*
**To L**	99.11 ± 6.65 *a*	51.92 ± 4.03 *a*,*b*	67.03 ± 3.26 *a*,*b*	53.62 ± 3.87 *a*
**Tp F**	83.89 ± 6.49 *a*	56.47 ± 3.05	67.92 ± 4.05 *a*,*b*	29.62 ± 2.63 *a*,*b*
**Vo R**	63.93 ± 3.97 *b*	52.54 ± 3.91 *a*,*b*	56.68 ± 3.12 *b*	25.33 ± 1.55 *a*,*b*
**Hp H**	108.04 ± 8.24 *a*	40.90 ± 2.75 *a*,*b*	83.20 ± 2.00 *a*,*b*	39.12 ± 2.97 *b*
**So R**	82.73 ± 6.65 *a*	52.89 ± 2.65 *a*,*b*	61.62 ± 3.75 *b*	37.43 ± 2.43 *b*
**Ps S**	90.11 ± 5.01 *a*	49.65 ± 2.75 *a*,*b*	57.90 ± 4.82 *b*	30.35 ± 2.69 *a*,*b*
**Lc S**	61.78 ± 4.60 *b*	32.46 ± 2.10 *a*,*b*	48.73 ± 4.28 *b*	30.22 ± 1.49 *a*,*b*

*a*: Statistically significant differences (*p* < 0.05) between the control group (C) and extracts. *b*: Statistically significant differences (*p* < 0.05) between the commercial biostimulant (CB) and extracts. Abbreviations: Ur L—*Urtica dioica* L. (leaves), Sg L—*Solidago gigantea* Aiton (leaves), To F—*Taraxacum officinale* F.H. Wiggers coll. (flowers), To L—*Taraxacum officinale* F.H. Wiggers coll. (leaves), Tp F—*Trifolium pratense* L. (flowers), Vo R—*Valeriana officinalis* L. (roots), Hp H—*Hypericum perforatum* L. (herb), So R—*Symphytum officinale* L. (roots), Ps S—*Pisum sativum* L. (seeds), and Lc S—*Lens culinaris* Medik. (seeds). Comparisons were made within each column.

**Table 5:** Antioxidant activity (DPPH, ABTS, and FRAP) (µM Trolox·g^−1^ FM) of seedlings (N = 4, mean ± SD).

	Without Stress			100 mM of Sorbitol			200 mM of Sorbitol			300 mM of Sorbitol		
Group	DPPH	ABTS	FRAP	DPPH	ABTS	FRAP	DPPH	ABTS	FRAP	DPPH	ABTS	FRAP
**C**	1.01 ± 0.04	8.68 ± 0.76 *b*	4.98 ± 0.09	0.29 ± 0.01	7.02 ± 0.37	5.54 ± 0.24	0.84 ± 0.05	3.29 ± 0.18	5.43 ± 0.11 *b*	0.74 ± 0.02	2.57 ± 0.11 *b*	4.41 ± 0.14
**CB**	1.10 ± 0.04	3.99 ± 0.27 *a*	5.09 ± 0.15	0.36 ± 0.02	7.63 ± 0.55	5.52 ± 0.08	0.80 ± 0.04	4.55 ± 0.36	4.71 ± 0.01 *a*	0.75 ± 0.05	9.96 ± 0.33 *a*	4.10 ± 0.12
**Ur L**	0.98 ± 0.06	9.00 ± 0.41 *b*	5.16 ± 0.10	0.53 ± 0.03 *a*,*b*	7.58 ± 0.32	5.67 ± 0.08	1.21 ± 0.04 *a*,*b*	5.80 ± 0.45 *a*	7.24 ± 0.23 *a*,*b*	0.24 ± 0.01 *a*,*b*	5.14 ± 0.42 *a*,*b*	4.31 ± 0.05
**Sg L**	0.98 ± 0.06	2.25 ± 0.13 *a*,*b*	5.12 ± 0.36	0.82 ± 0.05 *a*,*b*	9.41 ± 0.46 *a*	3.91 ± 0.13 *a*,*b*	0.75 ± 0.05	3.33 ± 0.10	5.64 ± 0.03 *b*	0.56 ± 0.04 *a*,*b*	5.18 ± 0.36 *a*,*b*	4.00 ± 0.06
**To F**	0.99 ± 0.05	3.04 ± 0.12 *a*	5.31 ± 0.11	0.39 ± 0.02	9.94 ± 0.44 *a*,*b*	4.59 ± 0.05 *a*,*b*	0.71 ± 0.04	5.18 ± 0.18	4.96 ± 0.07	0.61 ± 0.01 *a*,*b*	5.66 ± 0.35 *a*,*b*	4.98 ± 0.07 *a*,*b*
**To L**	1.46 ± 0.10 *a*,*b*	10.68 ± 0.50 *a*,*b*	8.24 ± 0.19 *a*,*b*	0.75 ± 0.04 *a*,*b*	6.08 ± 0.43	5.00 ± 0.22 *a*,*b*	1.28 ± 0.04 *a*,*b*	6.00 ± 0.24 *a*	6.80 ± 0.26 *a*,*b*	0.76 ± 0.05	6.17 ± 0.41 *a*,*b*	4.31 ± 0.08
**Tp F**	1.00 ± 0.04	9.00 ± 0.50 *b*	5.35 ± 0.22	0.75 ± 0.05 *a*,*b*	10.74 ± 0.27 *a*,*b*	4.23 ± 0.04 *a*,*b*	0.73 ± 0.05	4.76 ± 1.69	5.75 ± 0.08 *b*	0.20 ± 0.01 *a*,*b*	7.37 ± 0.49 *a*,*b*	3.58 ± 0.03 *a*,*b*
**Vo R**	1.17 ± 0.05	9.95 ± 0.60 *b*	4.71 ± 0.16	0.74 ± 0.06 *a*,*b*	10.42 ± 0.67 *a*,*b*	5.98 ± 0.16 *b*	1.09 ± 0.07 *a*,*b*	6.06 ± 0.09 *a*	9.33 ± 0.14 *a*,*b*	0.57 ± 0.04 *a*,*b*	5.52 ± 0.24 *a*,*b*	4.66 ± 0.25 *b*
**Hp H**	0.90 ± 0.05	14.25 ± 0.46 *a*,*b*	6.83 ± 0.16 *a*,*b*	0.62 ± 0.04 *a*,*b*	9.50 ± 0.72 *a*,*b*	5.75 ± 0.05	1.20 ± 0.06 *a*,*b*	5.45 ± 0.40 *a*	5.64 ± 0.31 *b*	0.56 ± 0.01 *a*,*b*	6.43 ± 0.47 *a*,*b*	5.90 ± 0.19 *a*,*b*
**So R**	1.12 ± 0.07	5.66 ± 0.34 *a*,*b*	5.25 ± 0.19	0.65 ± 0.04 *a*,*b*	10.71 ± 0.51 *a*,*b*	6.20 ± 0.12 *a*,*b*	0.87 ± 0.05	5.97 ± 0.29 *a*	6.77 ± 0.19 *a*,*b*	0.67 ± 0.04	8.04 ± 0.11 *a*,*b*	4.80 ± 0.11 *b*
**Ps S**	1.29 ± 0.07 *a*	15.42 ± 0.77 *a*,*b*	6.46 ± 0.13 *a*,*b*	1.07 ± 0.07 *a*,*b*	10.05 ± 0.38 *a*,*b*	4.75 ± 0.07 *a*,*b*	1.34 ± 0.07 *a*,*b*	6.26 ± 0.22 *a*	5.35 ± 0.15 *b*	0.66 ± 0.03	4.62 ± 0.34 *a*,*b*	4.29 ± 0.07
**Lc S**	1.14 ± 0.04	6.67 ± 0.17 *a*,*b*	6.41 ± 0.20 *a*,*b*	1.09 ± 0.06 *a*,*b*	10.06 ± 0.64 *a*,*b*	4.75 ± 0.04 *a*,*b*	0.78 ± 0.05	6.64 ± 0.28 *a*,*b*	5.97 ± 0.17 *b*	0.54 ± 0.03 *a*,*b*	7.18 ± 0.52 *a*,*b*	4.51 ± 0.05

*a*: Statistically significant differences (*p* < 0.05) between the control group (C) and extracts. *b*: Statistically significant differences (*p* < 0.05) between the commercial biostimulant (CB) and extracts. Abbreviations: Ur L—*Urtica dioica* L. (leaves), Sg L—*Solidago gigantea* Aiton (leaves), To F—*Taraxacum officinale* F.H. Wiggers coll. (flowers), To L—*Taraxacum officinale* F.H. Wiggers coll. (leaves), Tp F—*Trifolium pratense* L. (flowers), Vo R—*Valeriana officinalis* L. (roots), Hp H—*Hypericum perforatum* L. (herb), So R—*Symphytum officinale* L. (roots), Ps S—*Pisum sativum* L. (seeds), and Lc S—*Lens culinaris* Medik. (seeds). Comparisons were made within each column.

**Table 6:** The selected differentially expressed genes.

GenID	Gene Description	Log_2_FC	*p* Value	*p* adj
*Bol027348*	Chlorophyll A-B binding protein	−3.69133435	2.652 × 10^−119^	3.158 × 10^−115^
*Bol025609*	Haem peroxidase superfamily	−3.62134179	2.2754 × 10^−72^	3.6126 × 10^−69^
*Bol038846*	Chlorophyll A-B binding protein	−3.47106455	6.3414 × 10^−90^	2.517 × 10^−86^
*Bol035474*	Heat shock protein 70 family	−3.4314514	7.7544 × 10^−87^	2.3084 × 10^−83^
*Bol007329*	Heat shock protein 70 family	−3.30922549	1.0326 × 10^−62^	1.171 × 10^−59^
*Bol015841*	S-adenosylmethionine-dependent methyltransferases	−3.15086475	1.926 × 10^−104^	1.529 × 10^−100^
*Bol028754*	Pre-mRNA splicing Prp18-interacting factor	−3.14600571	5.6519 × 10^−38^	3.0743 × 10^−35^
*Bol030237*	Heat shock protein Hsp90 family	−3.05757472	3.128 × 10^−86^	8.277 × 10^−83^
*Bol042537*	Chlorophyll A-B binding protein	−3.03538031	8.6062 × 10^−80^	2.0496 × 10^−76^
*Bol022819*	GDSL lipase/esterase	−2.90676561	7.5131 × 10^−79^	1.6266 × 10^−75^
*Bol037866*	Chlorophyll A-B binding protein	−2.88826518	5.7314 × 10^−75^	9.7494 × 10^−72^
*Bol029651*	Glutathione S-transferase	−2.7703934	8.128 × 10^−175^	1.9357 × 10^−170^
*Bol031184*	Chlorophyll A-B binding protein	−2.72732175	1.1312 × 10^−65^	1.4179 × 10^−62^
*Bol027347*	Chlorophyll A-B binding protein	−2.66101166	2.484 × 10^−103^	1.479 × 10^−99^
*Bol024878*	Chlorophyll A-B binding protein	−2.64007581	2.4763 × 10^−87^	8.4247 × 10^−84^
*Bol008638*	Chlorophyll A-B binding protein	−2.5890437	7.2224 × 10^−71^	1.0118 × 10^−67^
*Bol037713*	Aldolase-type TIM barrel	−2.47272602	3.3404 × 10^−56^	3.1821 × 10^−53^
*Bol035765*	L-ascorbate oxidase	−2.46230838	1.6084 × 10^−61^	1.741 × 10^−58^
*Bol009860*	Chlorophyll A-B binding protein	−2.39712112	2.6808 × 10^−91^	1.2769 × 10^−87^
*Bol042916*	Chlorophyll A-B binding protein	−2.35392222	3.1169 × 10^−64^	3.7114 × 10^−61^
*Bol036973*	Chlorophyll A-B binding protein	−2.24533111	1.7584 × 10^−76^	3.2213 × 10^−73^
*Bol005916*	DnaJ Chaperone	−2.08718592	2.6651 × 10^−55^	2.3507 × 10^−52^
*Bol042486*	FKBP-type peptidyl-prolyl cis-trans isomerase	−1.79861464	5.3913 × 10^−72^	8.0246 × 10^−69^
*Bol039362*	Gibberellin regulated protein	−1.73887977	8.1552 × 10^−16^	7.4987 × 10^14^
*Bol036512*	Heat shock protein 70 family	−1.63213007	1.1957 × 10^−69^	1.5819 × 10^−66^
*Bol009568*	Heat shock protein Hsp90 family	−1.53770391	2.0328 × 10^−24^	4.655 × 10^−22^
*Bol028965*	DA1-like Protein	1.79625008	3.2363 × 10^−16^	3.1203 × 10^−14^
*Bol045470*	Calmodulin-binding protein 25	1.80170469	2.3543 × 10^−59^	2.4377 × 10^−56^
*Bol019811*	myb_SHAQKYF: myb-like DNA-binding domain, SHAQKYF class	1.86591201	1.3025 × 10^−26^	3.4852 × 10^−24^
*Bol034610*	RmlC-like cupin domain superfamily	2.04880192	6.8444 × 10^−36^	3.0185 × 10^−33^
*Bol007693*	B-box-type zinc finger	2.79507758	6.844 × 10^−58^	6.7912 × 10^−55^

## Data Availability

Data are contained within the article.
